# Tips for Conducting Telesimulation-Based Medical Education

**DOI:** 10.7759/cureus.12479

**Published:** 2021-01-04

**Authors:** Anita Thomas, Rebekah Burns, Elizabeth Sanseau, Marc Auerbach

**Affiliations:** 1 Pediatrics, Seattle Children's Hospital - Univeristy of Washington School of Medicine, Seattle, USA; 2 Pediatrics, Seattle Children's Hospital - University of Washington School of Medicine, Seattle, USA; 3 General Pediatrics: Emergency Medicine, Children's Hospital of Philadelphia, Philadelphia, USA; 4 Department of Pediatrics, Section of Pediatric Emergency Medicine, Yale University School of Medicine, New Haven, USA

**Keywords:** telesimulation, distance simulation, simulation in medical education, remote learning, healthcare simulation

## Abstract

Telesimulation utilizes communications technology, such as video conferencing platforms, to provide simulation-based medical education when participants and facilitators are geographically separated. Learners interact with each other, embedded participants, and a simulated patient and/or vital sign display on the computer screen. Facilitators observe the learners in real-time and provide immediate feedback during a remote debrief. Telesimulation obviates the need to have instructors, learners, and high fidelity patient simulators (HPS) in the same place, allowing simulation-based educational sessions to occur in institutions located remotely from simulation centers or when other barriers limit in-person education and/or training. For example, due to the novel coronavirus (COVID-19) pandemic, many medical education programs temporarily discontinued in-person simulations to adhere to physical distancing guidelines. The authors have reflected upon their experiences executing telesimulation sessions since the start of the pandemic and provide these 12 tips as practical suggestions on how to successfully implement telesimulations with medical trainees. These tips are intended to guide implementation and facilitation by staff and faculty trained in simulation.

## Introduction

The novel coronavirus (COVID-19) pandemic caused a significant impact on the education of medical students and trainees by limiting or altering both bedside and classroom-based learning [1,2]. For example, many medical simulation programs discontinued in-person simulations due to physical distancing requirements and shortages of personal protective equipment [3]. This necessary response has had a negative impact on learners across the continuum given that simulation-based medical education is a widely used, evidence-based educational method [4,5]. Simulation-based education is a core component of healthcare provider training and has been associated with improvements in health outcomes [6]. Gaps in training due to reduced access to simulation-based education could have long term consequences on health outcomes.

There is an increased demand for telesimulation in order to supplement trainee learning given that in-person education has been significantly restricted secondary to the COVID-19 pandemic [1]. Recently a taxonomy to define alternative simulation techniques has been created by the Society for Simulation in Healthcare [7]. Under this construct, telesimulation is defined as the employment of internet-based communication technology to provide simulation-based medical education where learners and instructors are located remotely from one another. The first reports of telesimulation described manikin-based simulation where the facilitator and/or simulation operations technologist remotely control simulation software in order to respond to participants’ interventions and lead the debriefing [8]. Given the concentration of trained facilitators at larger, often academic institutions, remote facilitation allows for the opportunity for teams to practice together while the technology is controlled from a separate site. Participants interact together with a simulator while facilitators in conjunction with simulation operations technical specialists, located remotely, control the manikin or other simulation technology, observe the exercise, and lead the debriefing using video conferencing technology [9]. This method requires that the participants have access to a high fidelity patient simulator (HPS) which can be operated remotely. Alternatively, a low fidelity model where vital signs and physical exam information are conveyed by the facilitator can be equally successful. This educational tool has been used to teach procedural, teamwork, and resuscitation skills across multiple disciplines, as well as assess learners remotely [10].

Telesimulation can also be performed with all learners and facilitators participating from physically distanced locations. Using a video teleconferencing platform such as Zoom (San Jose, CA), Microsoft Teams (Redmond, WA) or Skype (Palo Alto, CA), facilitators can share audio clips, still images, or pre-recorded videos, such as those hosted by American College of Emergency Physicians (ACEP) Simbox, to simulate a patient encounter [11]. This toolkit is free, open-access, and has pre-recorded videos of physical exam findings and patient monitors that facilitators can control according to scenario flow and participant interventions when conducting a simulation via a video conferencing platform. Facilitators acting as embedded participants fill the roles of caregiver (history provider) and bedside nurse who provides exam findings and enacts orders from the team. Learners can practice collecting history and requesting exam findings in an organized fashion. Clinical reasoning and teamwork/communication skills are practiced, just as they are in an in-person setting.

Regardless of technique, it is important to remember that healthcare simulation is grounded in Kolb’s experiential learning theory, where a concrete experience and active experimentation within a simulation allows for reflective observation and abstract conceptualization within the debriefing [12]. However, with telesimulation, the physical, hands-on aspect of participation may be limited, highlighting the need to adapt goals and objectives accordingly. While telesimulation simulation is still evolving and practices are being refined, these 12 tips summarize lessons learned from three different academic institutions that implemented remote telesimulation to maintain education goals in the setting of the COVID-19 pandemic.

## Technical report

Tip 1: Select a telesimulation technique that matches your resources.

Identifying resources available both within your institution and within the larger, global medical education community will influence which type of telesimulation you use with a particular group of learners. If an HPS is to be utilized, consider if the participants will be required to be present to interact with it while it is operated remotely. This will require adequate preparation of the simulation space before the learners arrive including ensuring that all needed supplies are present and that groups are small enough to comply with social distancing rules. A simulation technician may be required to operate the software remotely in addition to a minimum of one facilitator. Also, it is important to consider how embedded participants, such as a caregiver providing history, will interact with the team and what technology will be required to allow this to occur remotely. If using a simulated patient monitor and audio and visual files are used to create a telesimulation, additional equipment beyond a computer and internet connection is not required. A simulated monitor can be created via screen generator apps or software associated with an institution’s existing equipment and audio and visual files may be found online or created using images from actual patients or standardized patients. Multiple embedded participants are often preferred depending on learning objectives, and it may be challenging to engage more than five active participants during any given scenario.

Tip 2: Consider the limitations of telesimulation as compared to in-person simulation when choosing case topic and content.

When participants are not able to interact with an HPS in a face-to-face encounter, the ability to practice hands-on skills is severely limited, if not impossible. While participating remotely, learners are able to practice cognitive skills such as clinical reasoning as well as communication skills such as team communication and history taking but would not have the opportunity to practice hands-on-skills such as airway management or procedures. Therefore, cases that do not rely heavily upon procedure performance are more preferable for the format. If the telesimulation involves the use of audio and visual files, it is often difficult to demonstrate interventions and their results in real-time. For example, if a team requests that the embedded participant begins performing bag-mask ventilation on a patient, it may not be possible to show this in the ongoing video clip. The authors found that, if a scenario does require hands-on interventions such as CPR or bag-mask ventilation, having participants pantomime the actions to maintain visual cues for the entire team keeps the team immersed in the simulation while recognizing that this is insufficient for the actual practice of these skills.

If adapting simulations previously used for in-person simulation, consideration should be given to the number of critical actions required, as it is likely that fewer will be able to be achieved during a telesimulation, especially with all participants and facilitators joining remotely. Just as in face-to-face simulation encounters, a scenario could be broken down into “acts” with a debrief after a predefined set of actions are performed. Actors or facilitators portraying embedded participants may need to change their communication strategies to adapt to the video conferencing format, as well.

Tip 3: Identify learning objectives that are amenable to telesimulation.

As always, when preparing a simulation case, select two to three SMART (specific, measurable, attainable, relevant, time-bound) learning objectives. While these can often be adapted from in-person simulation scenarios, not all objectives will be achievable through this format if participants are participating remotely. For example, “Verbalize the need for peripheral access within two minutes of the simulation,” can be completed in person and via remote simulation, as it does not depend on participants being physically present for a simulation. However, a learning objective such as “Obtain a central line kit from the designated space in the intensive care unit within the first five minutes of the simulation” is not as translatable but could be adapted into “Describe where the central line kit would be found in the intensive care unit within the first five minutes of the simulation.” A skill-based objective such as “Perform high-quality two-person CPR within 10 seconds of a pulse check” may not be appropriate for a telesimulation if participants do not have access to adequate training resources. However, the objective could be altered to reflect knowledge of a skill even if performance cannot be achieved, for example, “Direct team to perform two-person CPR at a ratio of 15 compressions to two breaths." Facilitators should be mindful of selecting or modifying objectives when adapting existing in-person simulation scenarios to ensure that they are achievable during a remote simulation session.

Tip 4: Identify supplemental audio and visual materials to increase realism of patient evaluation.

As in in-person simulations, the use of supplemental audio-visual modalities can be helpful in enhancing realism. For example, backgrounds may be used on video conferencing platforms to mimic presence in the clinical space. Audio and visual cues can be shared by the facilitators to engage learners in the evaluation of the simulated patient. If the case involves a patient with a rash, for instance, incorporating a picture can allow participants to interpret the findings and incorporate this aspect into their diagnostic reasoning in a way that simply verbalizing that there is a rash will not. Audio clips of physical exam findings such as heart and lung sounds may also be used to force participants to make decisions based on their interpretation of auscultated clinical findings as opposed to reacting to a physical exam that is simply described to them. Videos demonstrating dynamic findings such as seizure or respiratory distress can allow participants to identify emergencies and help enhance the realism of the scenario. These supplemental materials, such as ACEP TeleSimBox, may help reinforce core concepts and challenge learners to apply higher-level reasoning [11]. Examples of free vital sign monitor simulators can be found at healthysimulation.com and Sourceforge.net.

Tip 5: Prepare your faculty and staff by piloting the telesimulation.

Given that this is a new modality for conducting simulations, the authors found it to be useful to trial individual telesimulations prior to rolling out with trainees. This allows facilitators to test all aspects of the scenario and may allow for the identification of technical difficulties. There are a variety of video conferencing modalities, each with pros and cons [13]. Running a trial of the functionality on whichever platform you choose to use allows facilitators to check each function, such as the chatbox or a breakout room. Trialing the telesimulation beforehand also allows the team to run through the scenario to troubleshoot any issues that come up, such as remote control of manikins and monitors and the timing of displaying labs or images. Collecting and acting on feedback from participants of the trial run allows for iterative improvements in the telesimulation.

Tip 6: Prepare the learners, including a pre-brief.

As telesimulation is a new modality for many learners and even facilitators, participants may benefit from an introductory email and will require a remote pre-brief before engaging in the scenario. In advance of the session, consider sending participants detailed instructions for using the video conferencing platform and expectations for the sessions. Depending on your learner group and goals, facilitators may wish to include preparatory materials related to the case topic such as review articles or relevant Free Open Access Medical Education (FOAMEd) posts [14].

The authors recommend using a scripted pre-brief at the start of each telesimulation. Participants and facilitators should introduce themselves and describe their overall role within the simulation (facilitator versus technician versus learner) as well as their clinical background and level of training. It is then important to review the traditional learning contract associated with simulation (i.e. that it is a safe and confidential space to learn) [15]. Next, the technical processes associated with telesimulation should be outlined. Acknowledging the limitations of the technology out loud (lack of physical presence, lack of visualizing the patient at all times, lack of normal visual cues from the team) is important so that participants can adequately frame the process, particularly for those who have previously participated in in-person simulations. Reviewing the expected use of features within the videoconferencing platform will allow participants to understand how to function within their roles. For example, describing how the chatbox or breakout rooms will be used to communicate with embedded participants or with the rest of the team will inform learners how to gather and/or record information during the simulation and prepare them for communication techniques that may be different from those applied in-person.

The authors also suggest using the pre-brief to confirm that everyone’s internet and software are working and that all participants can see the screen, chatbox, whiteboard, etc. Depending on the video conferencing modality, we also recommend that everyone keep their gallery/headshots minimized to allow for maximum viewing of the materials shared by the facilitators.

Tip 7: Allow for 'time outs'.

During the pre-brief, consider giving participants permission to call a 'time out' or 'huddle' during the telesimulation when needed for technological issues. The authors find that participants become frustrated and detracted from learning when challenges related to the equipment and software arise. Allowing them to take a pause to address these in real-time often allows them to re-engage in the scenario and continue. For example, a loudly beeping monitor may make it difficult for participants to hear one another and embedded participants. Allowing them to pause and request a change can help them refocus on the objectives of the session.

Tip 8: Establish team roles and communication strategies ahead of time.

It may be helpful to assign team roles during the pre-brief (or even in the introductory email before the simulation) in order to optimize the available time for active participation. Assigning three active participants as team lead, airway/survey provider, and history taker are often the minimum necessary to run a successful telesimulation. Additional participants could function as a recorder or act to identify clinical resources and lookup information requested by the team. Having critical information and actions recorded in the chatbox allows participants and facilitators alike to track what has happened, especially if individuals are periodically communicating in breakout rooms away from the rest of the team. We do recommend limiting the number of active participants to no more than four or five. If there are additional participants, they can be assigned to observe the scenario and provide feedback related to specific objectives or help keep track of critical actions and team communication for discussion during the debrief. Once roles are assigned all participants and facilitators should label themselves with their roles, if possible, on the screen. Remind non-active participants to turn their video off to minimize screen clutter, and participants should be instructed to hide or minimize non-video participants.

Due to the nature of videoconferencing, it is very difficult to have more than one person speaking at a time. To mitigate this challenge, facilitators can employ various functions within the software to allow participants to engage in their roles in parallel rather than in series. For example, the history taker may enter a “breakout room” with an embedded participant to gather history while the remainder of the participants and facilitators stay in the main “room” to manage the simulation patient. Alternatively, history can be collected in a written format in a chat box with the facilitator or live shared document. Physical exam findings might also be provided in this manner to minimize cross talk/audio interference and help maintain realism. The chatbox or whiteboard functions can be used to record data, as well.

It is also important to pre-emptively establish how facilitators will communicate with one another during the telesimulation. During in-person simulations, facilitators can directly talk to each other if the scenario needs to be modified in the moment or if confirmation that a critical action was completed is needed. This can be more difficult in a telesimulation as all verbal communication is shared amongst all participants. Thus, we suggest that facilitators establish a way to communicate beforehand, whether that is through the video conferencing platform private chat function, text messaging, or live shared document. This enables facilitators to maintain a line of communication separate from participants, should the need arise.

Tip 9: Prepare the debriefing environment.

As with in-person simulations, the debrief is one of the most important aspects of simulation [16,17]. Once the telesimulation has concluded, we recommend that the patient monitor screen be closed, and all participants turn on their video. This allows participants to view each other in a space separate from the simulation case, similar to how a debrief would be completed in person. Be sure to cover all phases of the debrief: reactions, description, analysis, and application to real-life practice [18]. In general, the debrief should be long enough to adequately review the simulated case and ensure that learning objectives have been addressed. However, given that telesimulation is novel to many institutions, we found it helpful to build in a little extra time during the debrief to allow for participants to give verbal feedback regarding the technical aspects of the case. Otherwise, maintain the debrief structure and content as close as possible to an in-person debrief.

Tip 10: Engage quiet participants.

Participants who speak up less often than their peers can be even less likely to speak up in video conference meetings [19]. This could be due to several factors including the need to take the extra step to unmute oneself, not having adequate privacy, or not wanting to interrupt someone else speaking. It can also be hard to participate in conversation when the normal visual cues are eliminated or distorted. Facilitators can call on such participants during the debrief, and even during the simulation, but it is important to let participants know they will do this during the pre-brief to maintain psychological safety. If participants were assigned the role of observer during the scenario, they should be invited to share their observations and feedback. Additionally, the authors suggest concluding debrief sessions by having each participant state one unique take-home point to apply in their future practice, thus engaging all participants one last time.

Tip 11: Share additional learning resources afterward.

Facilitators and participants can share learning resources via the share screen method during the debrief. We recommend that facilitators either create their own learning resource (e.g., a hand-out or slide-based presentation) or look to vetted FOAMed resources that can be easily shared. Given that participants are all located remotely, it is also helpful to share these resources electronically, either through the chat function, a learning management system, or by email for participants to review later if they wish.

Tip 12: Collect feedback to incorporate into the next telesimulation.

Verbal and written feedback is useful for the improvement of subsequent iterations of remote simulation. Soliciting verbal feedback is particularly helpful in telesimulation as it is an evolving modality. The authors suggest building an electronic simulation evaluation form to be completed after the remote simulation for participant self-assessment as well as to provide feedback to improve the simulation. The Evaluating Healthcare Simulation website has resources facilitators can access [20]. We recommend that this be accessed via QR code or link in the chatbox. Allowing for written, anonymous, and immediate feedback may provide facilitators with more clarity to improve the telesimulation.

## Discussion

In the midst of the COVID-19 pandemic and the need for physical distancing, telesimulation has become an increasingly common method of mitigating the lack of in-person simulation. As the pandemic restrictions are unpredictable, some institutions may rely on telesimulation to provide active education for the foreseeable future. Even in times of normal operation, telesimulation can be implemented in remote or low resource settings where facilitators, technicians, and simulation equipment may be limited. Adapting scenarios and learning objectives to minimize focus on hands-on procedural tasks and focus on communication and diagnostic reasoning is crucial for the creation of high yield telesimulations. Acknowledging the inherent challenges and limitations of this educational tool is an important aspect of orienting learners to the new learning environment and engaging them in sessions.

## Conclusions

While telesimulation does not serve as a perfect substitution of in-person simulation, in many cases, it can be used to actively engage participants in experiential learning and serve as an alternative when other options are not feasible. While this modality continues to be honed, the authors offer the above 12 tips developed from navigating telesimulations at their own institutions. Preparation and thoughtful consideration of objectives and methodologies paired with facilitated debriefing and simulation evaluation will help optimize successful implementation of this educational strategy.
